# FHL2 mediates podocyte Rac1 activation and foot process effacement in hypertensive nephropathy

**DOI:** 10.1038/s41598-019-42328-1

**Published:** 2019-04-30

**Authors:** Szu-Yuan Li, Pao-Hsien Chu, Po-Hsun Huang, Tsung-Han Hsieh, Katalin Susztak, Der-Cherng Tarng

**Affiliations:** 1 Division of Nephrology, Department of Medicine, Taipei Veterans General Hospital, Taipei, Taiwan; 2 Department of Medicine, National Yang-Ming University, Taipei, Taiwan; 3 Division of Cardiology, Department of Internal Medicine, Chang Gung Memorial Hospital, Chang Gung University, College of Medicine, Taipei, Taiwan; 4 Department of Critical Care Medicine, Taipei Veterans General Hospital, Taipei, Taiwan; 5 Joint Biobank, Office of human research, Taipei Medical University, Taipei, Taiwan; 6 Renal-Electrolyte and Hypertension Division of Department of Medicine, and Department of Genetics, Perelman School of Medicine, University of Pennsylvania, Philadelphia, USA; 7 Institute of Physiology, National Yang-Ming University, Taipei, Taiwan

**Keywords:** Stress fibres, Glomerular diseases

## Abstract

RAAS inhibition has been the standard treatment for CKD for years because it can reduce proteinuria and hence retard renal function decline, but the proteinuria reduction effect is still insufficient in many patients. Podocyte foot process and slit diaphragm are the final barrier to prevent serum proteins leak into urine, and podocyte foot process effacement is the common pathway of all proteinruic diseases. Cell structure are regulated by three evolutionarily conserved Rho GTPases, notably, Rac1 activation is sufficient and necessary for podocyte foot process effacement, however, Rac1 inhibition is not an option for kidney disease treatment because of its systemic side effects. Four-and-a-half LIM domains protein 2 (FHL2) is highly expressed in podocytes and has been implicated in regulating diverse biological functions. Here, we used micro-dissected human kidney samples, *in vitro* podocyte culture experiments, and a hypertension animal model to determine the possible role of FHL2 in hypertensive nephropathy. FHL2 was abundantly upregulated in hypertensive human glomeruli and animal kidney samples. Genetic deletion of the FHL2 did not alter normal renal structure or function but mitigated hypertension-induced podocyte foot process effacement and albuminuria. Mechanistically, angiotensin II-induced podocyte cytoskeleton reorganization via FAK-Rac1 axis, FHL2 binds with FAK and is an important mediator of Ang II induced Rac1 activation, thus, FHL2 inhibition can selectively block FAK-Rac1 axis in podocyte and prevent proteinuria. These results provide important insights into the mechanisms of podocyte foot process effacement and points out a promising strategy to treat kidney disease.

## Introduction

Renin–angiotensin–aldosterone system (RAAS) inhibition has been used to reduce proteinuria amount and delay renal function decline for decades, but the proteinuria reduction effect is still insufficient in many patients^1^. Studies revealed that the kidney expresses its own RAAS, inappropriate intra-glomerular RAAS activation has been found in many kidney diseases, including hypertensive nephropathy, diabetic nephropathy, and glomerulonephritis^2–4^. Amplifying RAAS signaling by overexpression of angiotensin II receptor on podocytes can directly induce foot process (FP) effacement, proteinuria, and glomerular sclerosis^5^, suggesting that the reno-protective effect of RAAS inhibition is mainly mediated by the podocyte. Podocytes are terminally differentiated cells with a complex cytoskeleton organization and unique protruding processes that turn into a characteristic interdigitating pattern with FPs of neighboring podocytes. These interlacing FPs are forming between the filtration slits that are bridged by the glomerular slit diaphragm (SD). Podocyte FPs and the interposed SD cover the outer part of the glomerular basement membrane (GBM) and plays a key role in establishing the selective permeability of the glomerular filtration barrier. The function of podocytes is based largely on their complex cell architecture. In most forms of kidney disease, injury to the podocyte results in a morphological change characterized by FP spreading and retraction by remodeling their cytoskeletal architecture during a process known as effacement^6^.

The most evolutionarily conserved Rho GTPases, including RhoA, Rac1, and Cdc42, are key determinants of actin polymerization that regulate a variety of cellular processes, such as adhesion, migration, division, and polarity. These Rho GTPases cycle between GTP and GDP-loaded activity states. After receiving signaling inputs, Rho-GTPases act through their effectors to polymerize and organize actin filaments into various configurations that change the cell shape. In the process of podocyte FP effacement, the bundled actin cytoskeleton of the foot processes is reorganized into broad membrane sheets that resemble the lamellipodia seen in cultured cells^7^. As in other cell types, Rho-GTPase and their regulators implicate dynamic shape changes in podocytes^8^.

Of the three Rho family GTPases, Cdc42 is critical for podocyte development. Podocyte-specific Cdc42 KO mice die by congenital nephrotic syndrome shortly after birth^9^. The other two Rho GTPases, Rac1 and RhoA, antagonize each other’s activation and function^10,11^. Rac1 plays a key role in actin lamellipodia induction and cell-matrix adhesion while RhoA is responsible for stress fiber formation. Recent studies revealed that overactivation of Rac1 is responsible for podocyte FP effacement and proteinuria^12–14^. Podocyte-specific expression of constitutively active Rac1 induces rapid FP effacement^7,14^. On the contrary, mice with podocyte-specific Rac1 deletion are protected from protamine induced FP effacement^15^. These studies suggest Rac1 activation is both necessary and sufficient for podocyte FP effacement. Many *in vivo* and *in vitro* studies showed Rac1 is regulated by FAK^16,17^, which receives signals from integrins and growth factor receptors, including angiotensin II receptor^18–20^,

The LIM domain is a cysteine-rich motif that has been proposed to direct protein-protein interaction. A diverse group of proteins containing LIM domains have been identified as displaying various functions. The functions of LIM domain proteins in the nucleus are mainly in gene regulation, whereas cytoplasmic LIM domain proteins are mainly involved in cytoskeleton organization^21^. Four-and-a-half LIM domains protein (FHL) contains exclusive four-and-a-half LIM domain–binding proteins. Proteins in this family function as adaptors or scaffolds to support the assembly of metameric protein complexes for critical cellular processes. The best-studied member of this family, FHL2, is highly expressed in cardiovascular system and kidney podocytes^22^.

In this current study, we explore the role of FHL2 in hypertensive kidney disease. We found FHL2 is upregulated in podocytes in hypertensive kidney disease samples. FHL2 interacts with FAK and is an important mediator of angiotensin II-induced Rac1 activation. Systemic FHL2 knockout mice are viable, maintaining normal kidney structure and function, but are protected from hypertension induced podocyte FP effacement and proteinuria. We also provide evidence that FHL2 levels are increased in subjects with different glomerular diseases other than hypertension, indicating that reduction of FHL2 may represent a novel therapeutic approach to kidney diseases.

## Results

### Podocyte FHL2 expression is upregulated in hypertensive kidneys

**Figure 1:**
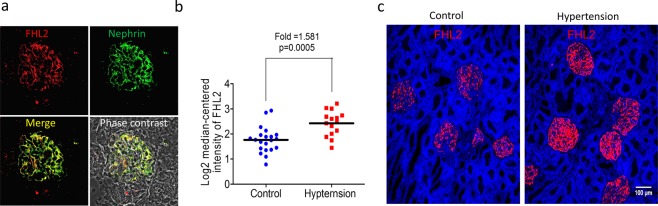
FHL2 is upregulated in human hypertensive kidney disease. Panel a: A representative double staining of FHL2 and the podocyte marker nephrin in human kidney. Panel b: FHL2 gene expression in micro-dissected human glomeruli. Panel c: Immune-fluorescent staining of FHL2 protein in control and hypertensive kidney disease samples, scale bar = 100 um.

Diabetic kidney disease and hypertensive nephropathy are the top leading causes of end-stage renal disease worldwide. Our previous study described FHL2 is abundantly expressed in podocyte cytosol and its nuclear translocation plays a critical role of Wnt pathway activation in diabetic nephropathy^22^. To study the role of FHL2 in hypertensive kidney disease, we first used immunofluorescence staining to survey FHL2 expression in normal kidney tissue, Fig. 1a illustrated FHL2 is expressed in human glomeruli and FHL2-positive cells were positive for podocyte marker nephrin. Next, we compared FHL2 expression in control and hypertensive kidney samples, we compared human glomerular microarray data from an international multicenter study, the European Renal cDNA Bank-Kroener-Fresenius biopsy bank. All kidney biopsies were micro-dissected and the disease etiology were stratified by the reference pathologists of the ERCB according to their histological diagnoses^23^. FHL2 expression in healthy control glomeruli (n = 21) were compared to samples obtained from hypertensive kidney disease patients (n = 15). As shown in Fig. 1b, FHL2 transcript level was significantly higher in hypertensive glomeruli (fold = 1.581, p = 0.0005). Protein expression was examined by immuno-fluorescent staining, agree with transcriptional changes, FHL2 protein expression is also higher in hypertension samples (Fig. 1c). Taken together, FHL2 expression is upregulated in podocytes in hypertensive kidneys.

### Ablation of FHL2 in mouse model of hypertension halts the development of proteinuria

**Figure 2:**
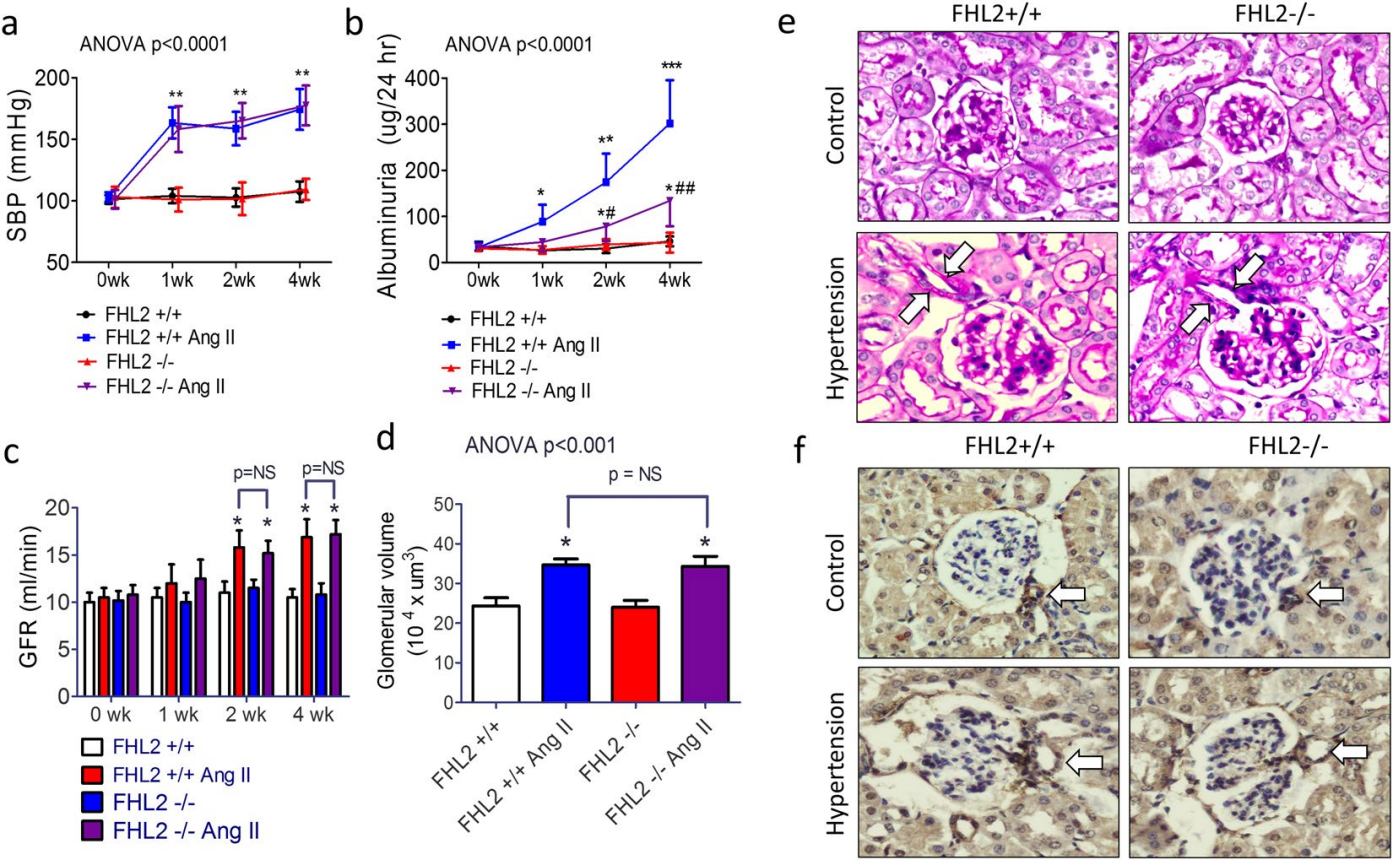
FHL2 knockout mice are protected from hypertensive proteinuria. Systolic blood pressure (panel a), 24 hour albuminuria (panel b), glomerular filtration rate (panel c) and glomerular volume (panel d) in hypertensive kidney disease mouse model. Panel e: Representative pictures of glomerular PAS stain. Panel f: Immuno-stain of α-SMA; Arrows: afferent arterioles. n = 8~10 each group. *p < 0.05 compared to normotensive WT group; **p < 0.01 compared to normotensive WT group; ***p < 0.001 compared to normotensive WT group; ^#^p < 0.05 compared to hypertensive WT group, ^##^p < 0.01 compared to hypertensive WT group.

To study the potential roles of the elevated FHL2 in hypertensive kidney disease, we used a continuous angiotensin II-induced hypertension model in FHL2 knockout mice and their wild-type littermates. Micro-osmotic pumps filled with angiotensin II were subcutaneously implanted in hypertension groups. Pumps filled with saline were implanted in control mice. To accelerate the development of hypertensive renal injury, 1% NaCl was added in drinking water in all groups as previous described^24^. Continuous angiotensin II-releasing pumps induced hypertension in mice within one week, as the hypertension status maintained throughout the whole study period. FHL2 deletion did not affect blood pressure at baseline or after angiotensin II treatment (Fig. 2a). We used metabolic cages to collect 24-hour urine weekly. In hypertensive groups, albuminuria started in the first week and gradually progressed. We found FHL2 knockout mice had significantly less albuminuria compared to their WT littermates at all time points (Fig. 2b). Mouse glomerular filtration rate (GFR) was calculated by FITC-labeled inulin as previous described^25^. In contrast to decreased creatinine clearance in CKD patients, we observed short-term angiotensin II infusion modest but significantly increase GFR in mice. FHL2 deletion does not affect GFR in baseline or after hypertension (Fig. 2c). Study animals were sacrificed 4 weeks after micro-osmotic pump implantation, at this time point, there were no difference of BUN and creatinine between the four groups. Pathological examination showed hypertensive kidneys have enlarged glomerular size (Fig. 2d,e) with afferent arteriole dilation and wall thickening (Fig. 2e,f). The mesangial cell expansion was mild and there was no difference between the two hypertensive groups. We did not observe tubular-interstitial fibrosis, glomerular sclerosis in kidney sections, or qPCR gene expression analysis in this model (data not shown). In summary, continuous angiotensin II infusion induced arterial hypertension, glomerular hyper-perfusion, and albuminuria in mice. Although had similar severity of hypertension and hemodynamic changes, FHL2 knockout mice were protected from hypertension induced albuminuria.

### Angiotensin II-induced podocyte cytoskeleton remodeling via FAK-Rac1 axis

The results of animal experiment led us to study the mechanism of angiotensin II-induced glomerular permeability changes. Since WT hypertensive mice developed albuminuria and we did not observe glomerular histological difference under light microscopy, we speculated that angiotensin II might change podocyte ultrastructure and FHL2 is involved in this process.

**Figure 3:**
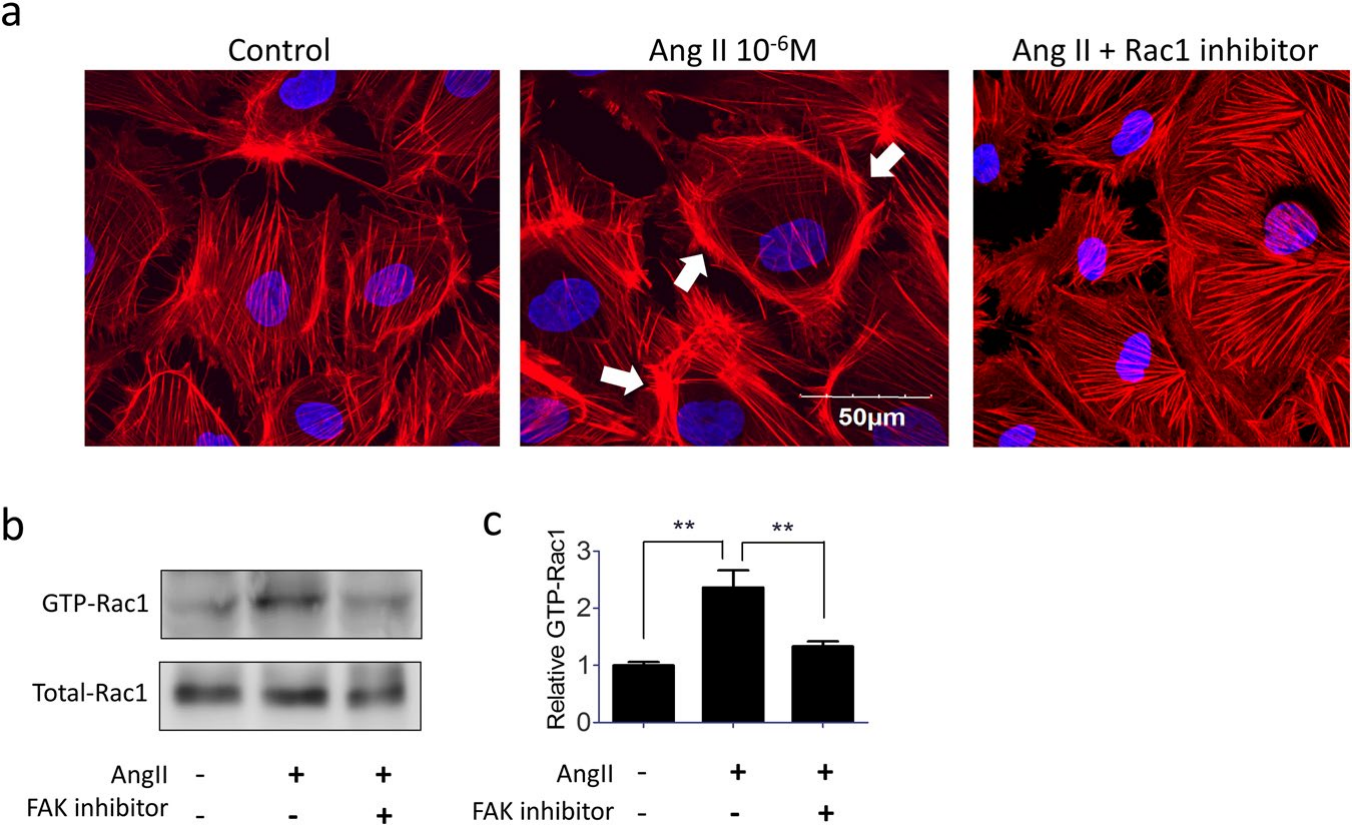
Angiotensin II stimulates podocyte cytoskeleton rearrangement through FAK-Rac1 pathway, Angiotensin II stimulates podocyte cytoskeleton rearranged to marginal edge of the cell and form lamellipodium (arrows). This altered cytoskeleton pattern can be prevented by selective Rac1 inhibitor (Panel a). Angiotensin II-induced Rac1 switch-on can be blocked by FAK inhibitor (Panel b). Panel c: Quantification of active Rac1 (n = 3). **p < 0.01 compared to controls.

In cultured podocytes, angiotensin II treatment induced cytoskeleton remodeling to the edge of the cell (lamellipodium) (Fig. 3a). Since FAK/Rac1 signaling has been documented to control lamellipodium formation and cell migration in many cell types^16,26^, we tested the role of FAK/Rac1 in angiotensin II-induced cytoskeleton remodeling in podocytes. As shown in Fig. 3a, angiotensin II-induced podocyte cytoskeleton reorganization can be blocked by selective Rac1 inhibitor, this result is consistent with previous animal experiment suggesting Rac1 activation is a necessary step of FP effacement^7^. Because FAK is an upstream regulator of Rac1 in other cell type^16^, and podocyte-specific FAK KO can protect mice from LPS induced FP effacement and proteinuria^27^, we therefore hypothesized that FAK could also activate Rac1 in podocytes. Using small GTPase pull-down assay and immunoblotting, we found angiotensin II treatment increase GTP-Rac1 expression in podocytes, and this change can be blocked by FAK inhibitor (Fig. 3b,c). These data confirmed angiotensin II-induced podocyte cytoskeleton reorganization (lamellipodium) is mediated by the FAK-Rac1 axis.

### FHL2 is an important scaffold protein of Fak-Rac1 axis activation in podocyte

**Figure 4:**
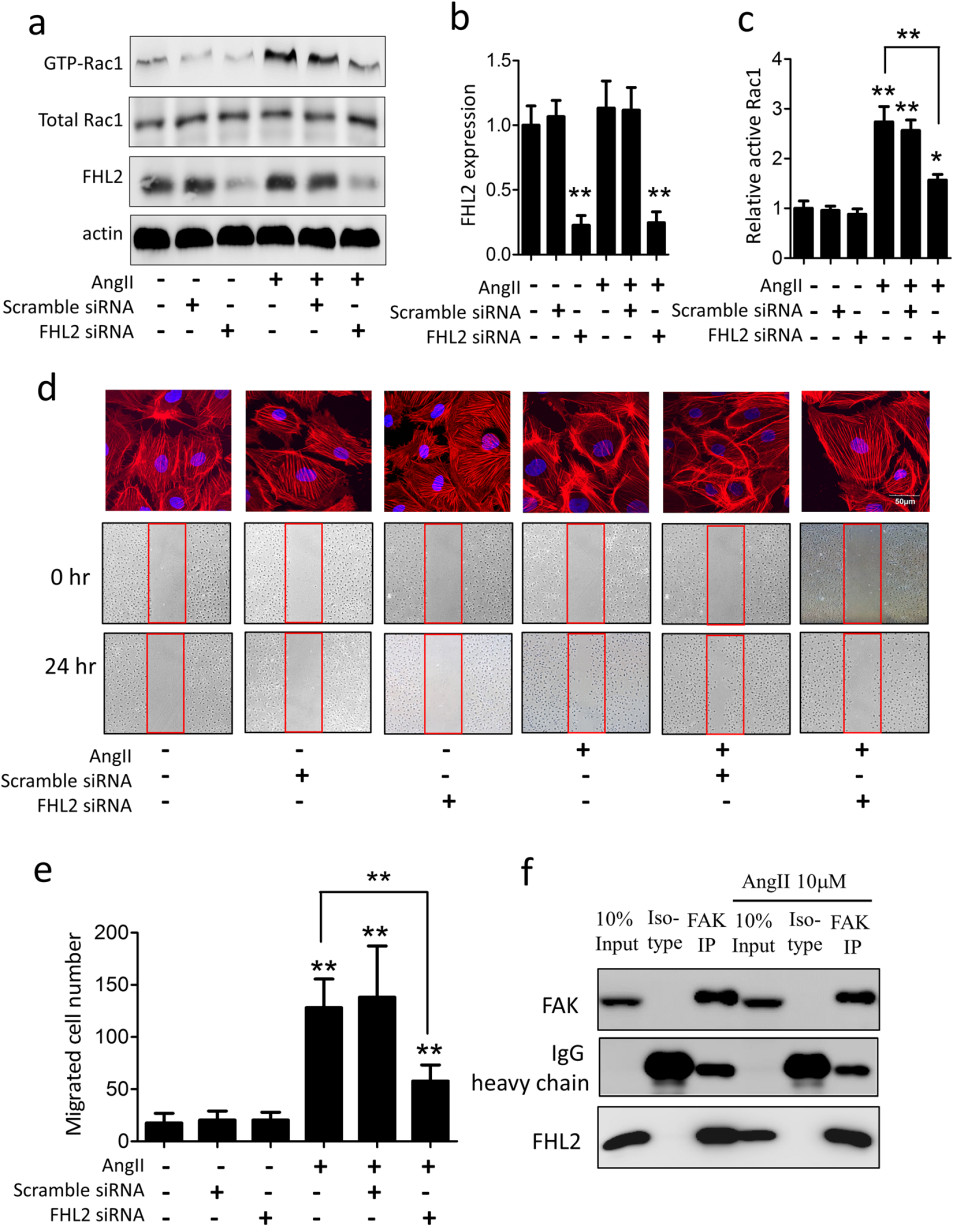
knockdown FHL2 prevents angiotensin II-induced Rac1 activation and cytoskeleton rearrangement in podocytes *in vitro*. Panel a: A representative western blot of FHL2, active- and total Rac1 expression after angiotensin II treatment in cultured podocytes. Panel b: Quantification of FHL2 expression (n = 3). Panel c: Quantification of active-Rac1 (n = 3). Panel d: Representative figures of podocytes lamellipodium formation and migration. Panel e: Quantification of migrated podocyte number (n = 3). Panel f: Co-immunoprecipitation (Co-IP) confirmed FHL2 and FAK has structural protein-protein interaction in podocytes. *p < 0.05, **p < 0.01 compared to control.

To study the role of FHL2 in podocyte cytoskeleton remodeling, we knock down FHL2 in cultured podocytes and treated cells with angiotensin II. Unlike the FHL2 upregulation in hypertensive kidneys *in-vivo*, we didn’t observe angiotensin II changes FHL2 expression in cultured podocytes (Fig. 4a,b). Knockdown FHL2 did not alter baseline Rac1 activity but significantly attenuated angiotensin II-induced Rac1 activation (Fig. 4a,c). To further confirm the effect of FHL2 in podocyte cytoskeleton reorganization, we performed wound scratch assay as previous described^27^. Without angiotensin II treatment, knockdown FHL2 did not alter podocyte cytoskeleton structure or cell migration. Angiotensin II treatment remarkably increased lamellipodium formation, and these cells were more migratory. We found FHL2 knockdown significantly attenuated angiotensin II-induced cytoskeleton changes, as well as the migratory phenotype (Fig. 4d,e). We next used CO-IP strategy to screen Rac1 upstream molecules and found FHL2 and FAK has structural protein-protein interaction in podocytes, regardless of angiotensin II presence (Fig. 4f). In summary, FHL2 has protein-protein interaction with FAK in podocytes, although this interaction has been observed in other cell types with different biological function^28,29^, its role in podocyte disease was unknown. Our functional studies illustrated FHL2 is an important mediator of angiotensin II-induced Rac1 activation, and FHL2 inhibition can effectively block angiotensin II-induced cytoskeleton reorganization in podocytes.

### FHL2 knockout prevents podocyte foot process effacement in hypertensive animals

**Figure 5:**
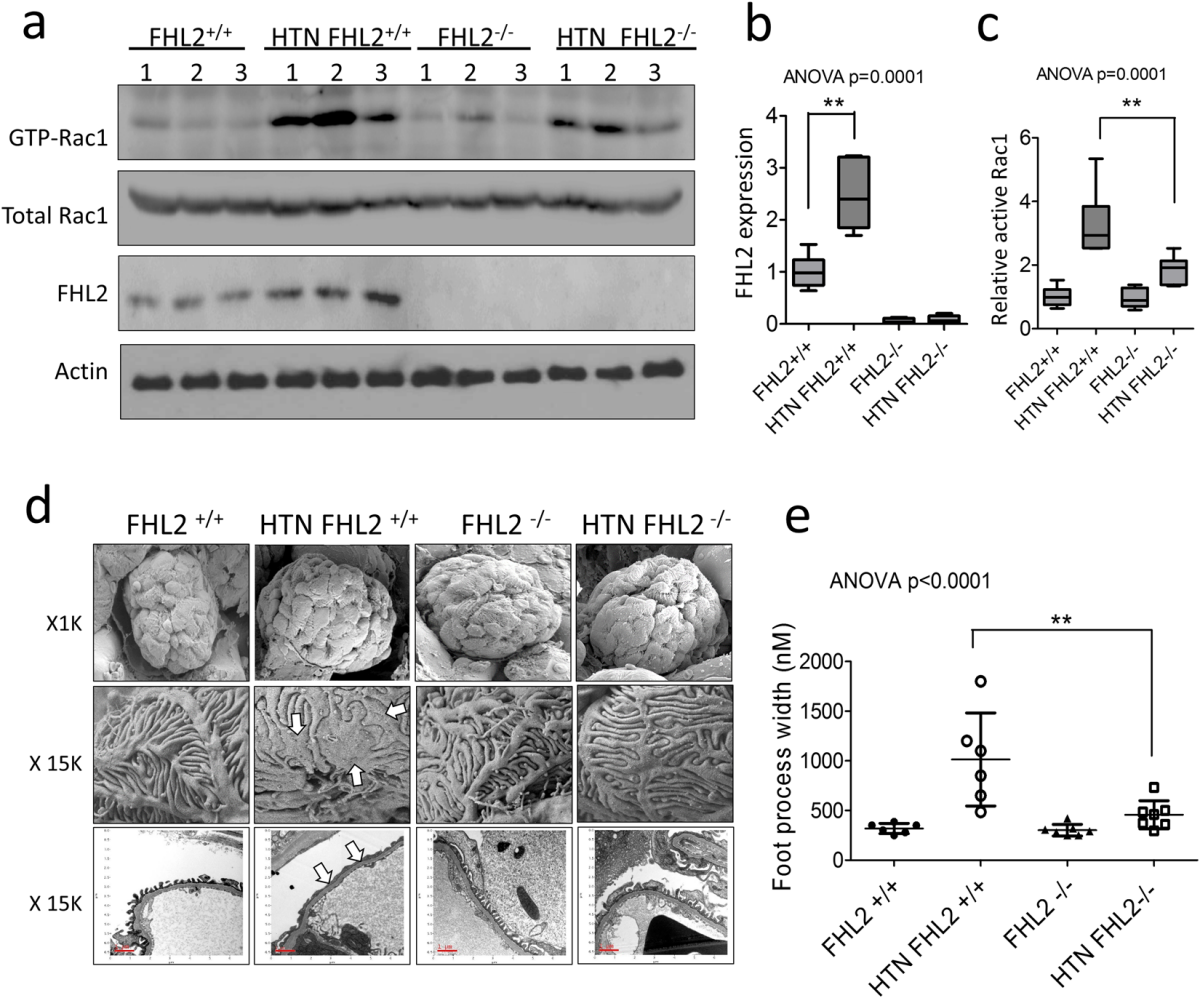
FHL2 ablation prevents hypertension induced Rac1 activation and podocyte FP effacement *in vivo*. Panel a: Protein expression of Rac1 and FHL2 in mouse glomeruli. Panel b and c: Quantification of FHL2 and active-Rac1. Panel d: Scanning and transmission electron microscopy examination of the mouse glomeruli, the interdigitating podocyte foot process became widened and flattened in hypertensive WT mice (arrows), and FHL2 knockout prevented mice from these podocyte ultrastructure changes. Panel e: Quantification of foot process effacement. n = 6~7 each group. **p < 0.01.

Since *in vitro* experiments suggest FHL2 facilitate FAK activity to turn-on Rac1 in hypertensive podocytopathy, we therefore isolated glomeruli from the four groups of mice to analyze Rac1 activity *in vivo*. In line with human data, we found glomerular FHL2 expression is higher in hypertensive mice (Fig. 5a,b). We also confirmed hypertensive WT glomeruli have higher expression of GTP-Rac1 (Fig. 5a,c). Normotensive FHL2 KO mice have unchanged baseline Rac1 activity, but the upregulation of GTP-Rac1 level was significantly suppressed in hypertension compared to their hypertensive WT littermates (Fig. 5c). Under electron microscopic exam, hypertensive mice have bigger glomerular size where their foot processes became widened and effaced (Fig. 5d). We did not observe EM evidence of GBM thickening or endothelial injury in this model. Consistent with Rac1 activity and albuminuria amount, podocyte FP was much less in hypertensive FHL2 KO group (Fig. 5e). In summary, these data further confirmed the role of FHL2 in podocyte Rac1 activation and FP effacement *in vivo*.

## Discussion

**Figure 6:**
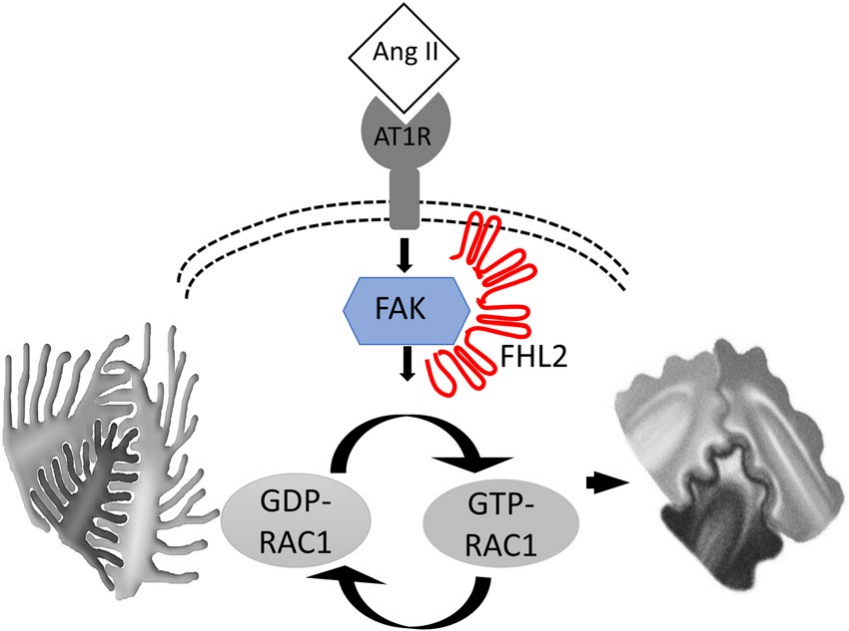
Purposed model of FHL2 in podocyte cytoskeleton dynamic regulation. Pathological stimuli (i.e. angiotensin II) activates FAK to switch GDP-RAC1 (inactive form) to GTP-RAC1 (active from), causing podocyte cytoskeleton reorganization and FP effacement. FHL2 has protein-protein interaction with FAK and mediates Rac1 activation. FHL2 inhibition prevents podocyte Rac1 activation and foot process effacement.

In this current study, we reported FHL2 is an important mediator in hypertensive nephropathy. In diseased kidneys, angiotensin II stimulates FAK to switch-on Rac1, which is a critical step to initiate FP effacement. We analyzed micro-dissected human glomeruli gene expression and used immunostaining to illustrate FHL2 is upregulated in hypertensive podocytes. We also showed FHL2 has direct protein-protein interaction with FAK and is an important cofactor of Rac1 activation in podocytes. FHL2 is dispensable for normal podocyte function, but FHL2 inhibition can block Rac1 activation and prevent podocyte FP effacement (Fig. 6). Although the upregulation of FHL2 is evidenced in clinical data and the hypertensive animal model, we didn’t observe angiotensin II changes FHL2 expression in cultured podocytes, one explanation is that angiotensin II itself is not a FHL2 stimulator, but its downstream effectors promote FHL2 upregulation *in-vivo*. For example, Ang II stimulates TGF-β expression in the kidney cells^30,31^ and TGF-β is a very strong FHL2 stimulator in podocytes^22^.

Proteinuria is the first clinical sign of kidney disease, although RAAS inhibition can reduce proteinuria, the result is not always satisfying. Dual blockade of ACEI/ARB even increases the risk of adverse effects^32–34^. A more specific and precise intervention is therefore required. Overactivation of Rac-1 has been shown as the corpus of podocytes FP effacement in human diabetic nephropathy, hypertensive kidney disease and nephrotic syndrome^13,35^. Glucocorticoid therapy has been used to treat nephrotic syndrome for decades, and a recent study using live-cell imaging finally revealed that steroids act directly on the podocyte to reduce Rac1 activity^36^. Another validation on the importance of Rac1 was the discovery of human mutations in *ARHGDIA*, which resulted in steroid-resistant nephrotic syndrome^37^. In mice lacking *Arhgdia*, podocyte FP effacement and proteinuria were associated with increased Rac1 (but not RhoA)^37^. These reports all suggest Rac1 inhibition could be a very powerful intervention for proteinuria kidney diseases^37,38^. However, Rac1 is ubiquitously expressed in many cell types and regulates complex cell functions, systemic Rac1 inhibition could therefore alter normal cell biology and cause severe side effects^39–41^. To the best of our knowledge, there is no pharmacological intervention can specifically inhibit Rac1 activity in the kidney yet.. In the current study, we illustrated FHL2 has protein-protein interaction with FAK, the main upstream activator of Rac1. *In-vivo* and *in-vitro* experiments showed FHL2 inhibition prevent podocyte Rac1 activation without altering normal biological function, pointing out FHL2 inhibition is a good alternative of systemic Rac1 inhibition in treating kidney diseases.

FHL2 has been identified as scaffold in diverse protein complexes of multiple signaling pathways. FHL2 has protein interaction with beta-catenin^22^, AP1^42^ and androgen receptor^43^ and involves in integrin other signal transmission^44,45^. Gabriel *et al*. first observed FHL2 and FAK form a protein complex in ovary carcinoma^28^, in this current study, we extended this protein-protein interaction to podocytes. Since FAK^46^ and FHL2^28,47–49^ are upregulated in several types of cancers^28,47–49^, further studies are needed to test whether FHL2 mediate tumor cell invasion in these malignancy.

As Rac1 activation is the common pathway of podocyte effacement, FHL2 inhibition therefore should be effective not only in hypertensive nephropathy but also in other glomerular diseases. We have analyzed FHL2 expression level in human glomerular samples and found it is up-regulated in almost all kind of proteinuric kidney diseases, including hypertension, diabetes, FSGS, membranous nephritis, IgA nephropathy, and nephritis caused by lupus or vasculitis (Supplement Fig. 1). To test whether FHL2 inhibition can prevent proteinuria in these diseases, we introduced Adriamycin-induced FSGS model in mice and found FHL2 KO animals developed less albuminuria than their WT littermates (Supplement Fig. 2). Although further studies are needed to test the renoprotective effect in other types of kidney disease, results from hypertension, FSGS and diabetes animal models^22^ support FHL2 inhibition could be a universal therapeutic strategy to treat glomerular diseases.

In summary, our results showed FHL2 has protein-protein interaction with FAK in podocytes, Inhibition of FHL2 can prevent Rac1 activation and protect mice from podocyte foot process effacement and proteinuria. An advantage of FHL2 inhibition is that systemic FHL2 gene knockout mice doesn’t develop side effects after long-term follow up, suggesting FHL2 inhibition is a safe and effective intervention to treat kidney diseases.

## Methods

### Glomeruli gene expression analysis and immunostaining in human samples

Human kidney specimens were obtained from diagnostic renal biopsies and nontumor kidney tissue from patients with renal malignancy and nephrectomy. Human glomerular microarray data was accessed from an international multicenter study, the European Renal cDNA Bank-Kroener-Fresenius biopsy bank. Biopsies were obtained from patients after informed consent and with approval of the local ethics committees. Glomeruli were isolated using manual microdissection in human kidney samples. Microarray data from hypertensive kidney disease samples (GSM920386-920400) and normotensive healthy controls (GSM807930-807931, 807934, 920368–920385) were compared^23^. Glomerular gene expression profiles were obtained from GEO; http://www.ncbi.nlm.nih.gov/geo under accession number GSE47185. The data was processed using Bioconductor R package. To check FHL2 protein expression in normotensive and hypertensive kidney, samples were double-stained with FHL2 and a podocyte marker Nehrin. The images were all taken under same laser power and gain. The experiments were carried out in accordance with relevant guidelines and regulations. Taipei Veterans General Hospital Institutional Review Board approved the study. All enrolled participants gave their informed consent.

### Hypertension kidney injury model

To induce hypertension, Alzet osmotic minipumps (model 2004; ALZET Scientific Products, Mountain View, CA, USA) were subcutaneously implanted into 10-week-old FHL2-/- and their FHL2 + / + littermates (C57BL/6 bacfkground, all male)^22^. Before implantation, osmotic mini-pumps were filled with solutions of angiotensin II (Sigma Chemical CO., St Louis, MO, USA) that delivered 1000 ng/kg/min in hypertension groups or saline in control groups^50^. Blood pressure was determined prior to the implantation and then weekly during the study period. Blood pressure was measured in conscious mice using a tail-cuff apparatus (Visitech BP-2000 Bloos Pressure Analysis System). Mice were initially acclimated to the device for two consecutive days before the formal measurement. 24-hour urine was collected by metabolic cages weekly. For measurement of urine albumin, the 24-hour urine was centrifuged and albumin level was determined using a commercialized ELISA (Albuwell M kit; Exocell Inc). Mouse GFR was assessed by the FITC-inulin clearance method as previously described^25^. At the end of the study, mice were euthanized and kidneys were removed for analysis. All experimental protocols and procedures were performed in accordance with the relevant guidelines and regulations and approved by the institutional animal care committee of Taipei Veterans General Hospital (IACUC 104-095).

### Podocyte cell culture

The conditional immortalized human podocyte line was cultured as previously described^22^. Briefly, cells were cultured at 33 °C in RPMI 1640 medium supplemented with 10% FBS and 1% ITS (Sigma-Aldrich, St. Louis, MO). To induce differentiation, podocytes were moved to 37 °C. All experiments were performed 14 days after thermoshift. Cultured podocytes were treated with angiotensin II (10^−6^ M; Sigma-Aldrich) to mimic the cytoskeleton changes *in vivo*. To study the podocyte cytoskeleton regulation mechanism, cells were pre-treated with Rac1 specific inhibitor (NSC2376650 μM; Sigma-Aldrich) or FAK specific inhibitor (FAK 14 CAS 4506-66-5, 10uM Sigma-Aldrich) for 30 minutes before angiotensin II stimulation. For some experiments, scrambled siRNA or FHL2 siRNA (Santa Cruz Biotechnology) were transfected by Lipofectamine 3000 (ThermoFisher).

### Podocyte cytoskeleton rearrangement and cell migration

To detect cell cytoskeleton rearrangement, podocytes were cultured on sterile coverslips. After angiotensin II stimulation, cells were fixed with 4% paraformalin for 15 min and cell membranes were permeabilized by 1% Triton X-100 for 3 min. Fluorescent-labelled phalloidin was used to study the distribution of actin filaments in cells^51^. All slides were viewed and recorded under an Olympus FV10i confocal microscope, which is capable for a high throughput scanning of every cell on a slide. Since cell membrane and cytoskeleton protrusions at the leading edge of cells, known as lamellipodia, drive cell migration, we used a P1000 pipette tip to create a straight “wound” in a podocyte monolayer in 6-well plates to objectively quantify the migration of cells. Images were captured at the beginning and the 24-hour mark, the number of migrated cells were counted by Image-J software.

### Small GTPase pull-down assays and Co-IP

The active RAC1 pull-down kit (Pierce Biotechnology Rockford, IL) was used to assay the active form of RAC1. Briefly, Glutathione S-transferase (GST) conjugated human pak1-PBD was added to the cell extracts to pull down the active form of RAC1. The active form of RAC1 (GTP-Rac1) was eluted, subjected to western blot analysis, and the expression of total RAC1 was used as control. Immunoreactive bands were visualized by an enhanced chemiluminescence reaction with an ECL Prime Western Blot Detection System). The GTP-Rac1, FHL2 and actin western blots were cropped from different parts of the same gel, and the total Rac1 were from corresponding gels. Intensity of the chemiluminescent bands was quantitatively analyzed by Image J (NIH).

For coimmunoprecipitation experiments, podocytes were cultured in RPMI with or without angiotensin II. Endogenous FHL2 and FAK were coimmunoprecipitated by a commercial kit (Pierce Biotechnology) according to the manufacturer’s protocol. Anti-FHL2 antibody was purchased from MBL Life Science (Nagoya, Japan). Anti-FAK antibody was purchased from Abcam (USA).

### Scanning and Transmission Electron Microscopy

The three-dimensional ultrastructure of mouse glomeruli were studied by scanning electron microscopy using standard protocol. Briefly, kidney cortex was cut into small pieces and fixed with 2.5% glutaraldehyde followed by 1% osmium tetroxide. Samples were dehydrated in a graded series of ethanol (to 100%) and critical point dried. The specimens were freeze-fractured, and sputter coated with platinum. Samples were observed and recorded in a JSM-7600F (JEOL) scanning electron microscope. For TEM examination, specimens were series dehydrated and embedded into epoxy resin according to routine procedures. Ultrathin sections were stained with 2% uranyl acetate and 1% lead citrate. Sections were viewed and recorded using a JEM-1400 plus microscope. Foot process width was measured under 15,000x magnification. Five glomeruli were randomly selected in each mouse, and the width of 50 continuous foot processes were then measured to get an average number of each glomeruli.

### Statistics

Statistical analyses were performed using GraphPad Prism software. All values are expressed as mean and standard deviation or box-and-whisker plots. A 2-tailed Student’s t test was used to compare 2 groups. One-way ANOVA with post-hoc Tukey test was used to compare multiple groups. A P-value less than 0.05 was considered statistically significant.

## Supplementary information

Supplement
