# Author Correction: FHL2 mediates podocyte Rac1 activation and foot process effacement in hypertensive nephropathy

**DOI:** 10.1038/s41598-019-51739-z

**Published:** 2019-10-24

**Authors:** Szu-Yuan Li, Pao-Hsien Chu, Po-Hsun Huang, Tsung-Han Hsieh, Katalin Susztak, Der-Cherng Tarng

**Affiliations:** 1 Division of Nephrology, Department of Medicine, Taipei Veterans General Hospital, Taipei, Taiwan; 2 Department of Medicine, National Yang-Ming University, Taipei, Taiwan; 3 Division of Cardiology, Department of Internal Medicine, Chang Gung Memorial Hospital, Chang Gung University, College of Medicine, Taipei, Taiwan; 4 Department of Critical Care Medicine, Taipei Veterans General Hospital, Taipei, Taiwan; 5 Joint Biobank, Office of human research, Taipei Medical University, Taipei, Taiwan; 6 Renal-Electrolyte and Hypertension Division of Department of Medicine, and Department of Genetics, Perelman School of Medicine, University of Pennsylvania, Philadelphia, USA; 7 Institute of Physiology, National Yang-Ming University, Taipei, Taiwan

Correction to: *Scientific Reports* 10.1038/s41598-019-42328-1, published online 30 April 2019

This Article contains errors in Figure 4, where the incorrect images were used for podocyte migration in Figure 4d, which also affected Figure 4e. The correct Figure 4 appears below.

**Figure 4:**
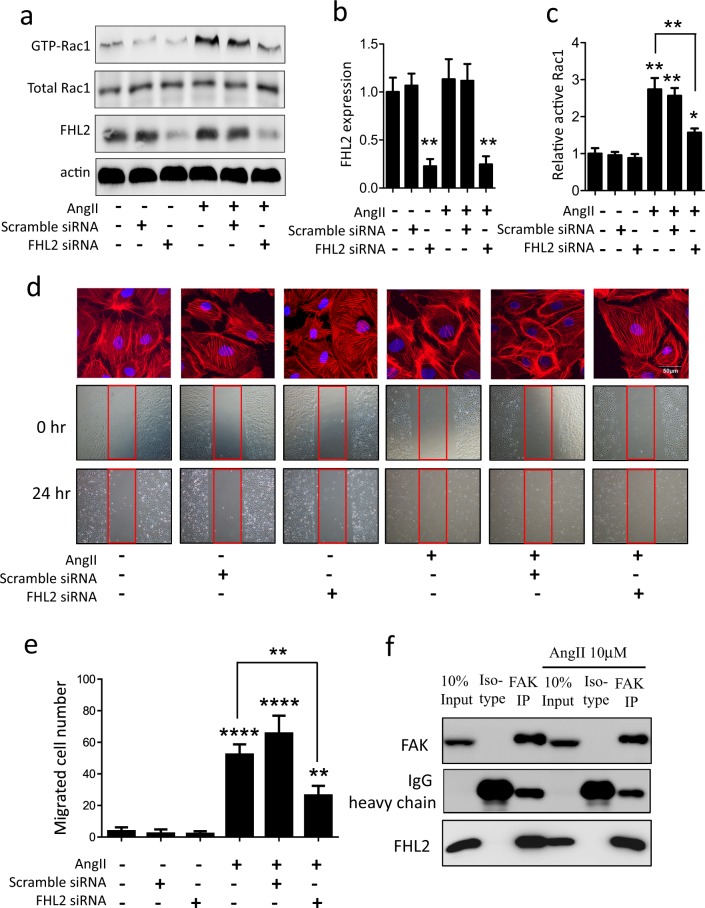
.

The main conclusions of the Article are unaffected by these changes.

