# Supplementary material for: FHL2 mediates podocyte Rac1 activation and foot process effacement in hypertensive nephropathy
Source: Sci Rep. 2019 Apr 30;9:6693. doi: 10.1038/s41598-019-42328-1 (PMC6491468; doi:10.1038/s41598-019-42328-1)

FHL2 mediates podocyte Rac1 activation and foot process effacement in hypertensive nephropathy

Szu-Yuan Li , Pao-Hsien Chu , Po-Hsun Huang , Tsung-Han Hsieh, Katalin Susztak, Der-Cherng Tarng

Supplement Figure 1. FHL2 is up-regulated in different type of glomerular diseases.

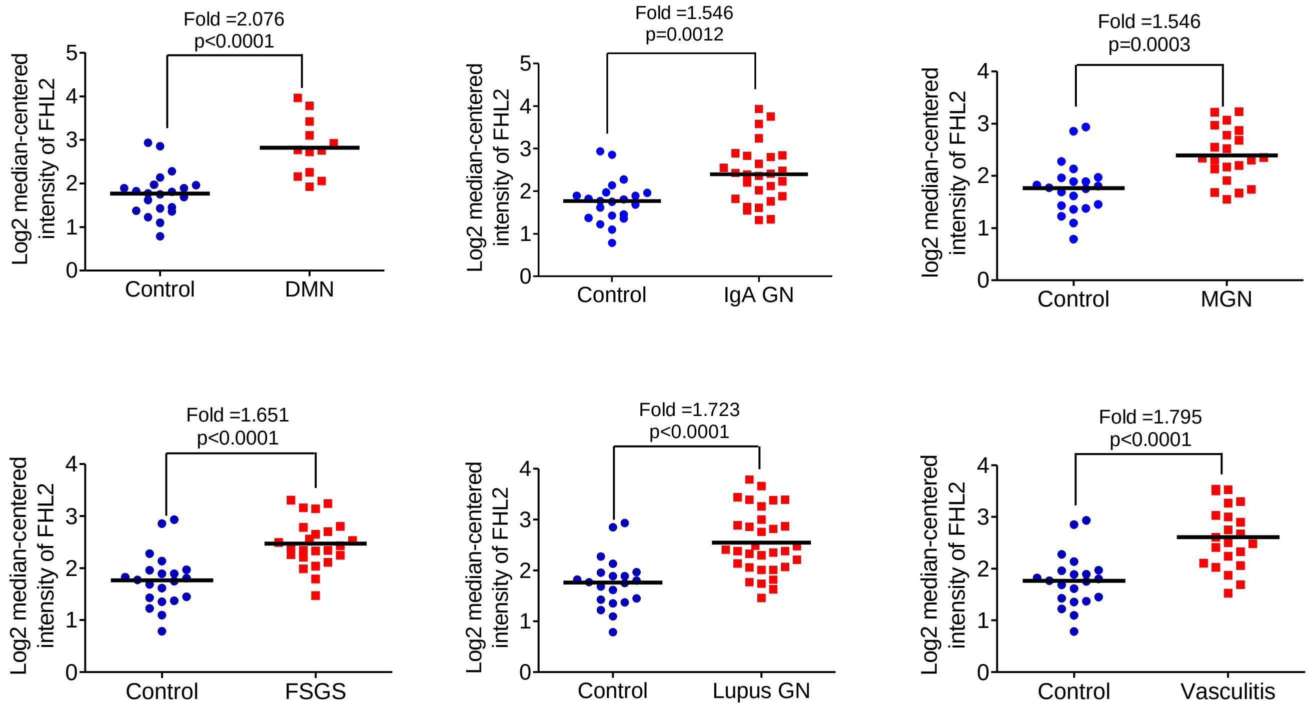

FHL2 expression in micro-dissected human glomerular samples. DMN: DM nephropathy; IgA GN: IgA nephropathy; MGN: membranous glomerulonephritis; FSGS: focal segmental glomerulosclerosis; lupus GN: lupus glomerular nephritis.

Supplement Figure 2. FHL2 KO mice developed less albuminuria in adriamycin-induced FSGS

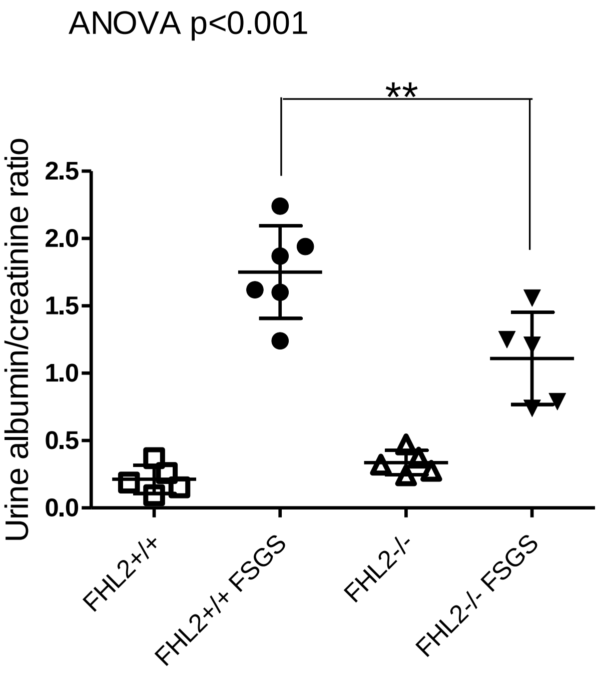

Spot urine albumin/creatinine ratio in four group of mice. Saline or Adriamycin (25mg/Kg) were tail vein injected into Control and FHL2 knockout mice. Urine was collected 1 week after injection. n=5~6 each group, ** $p<0.01$

Uncropped western blot gels in figure 4a

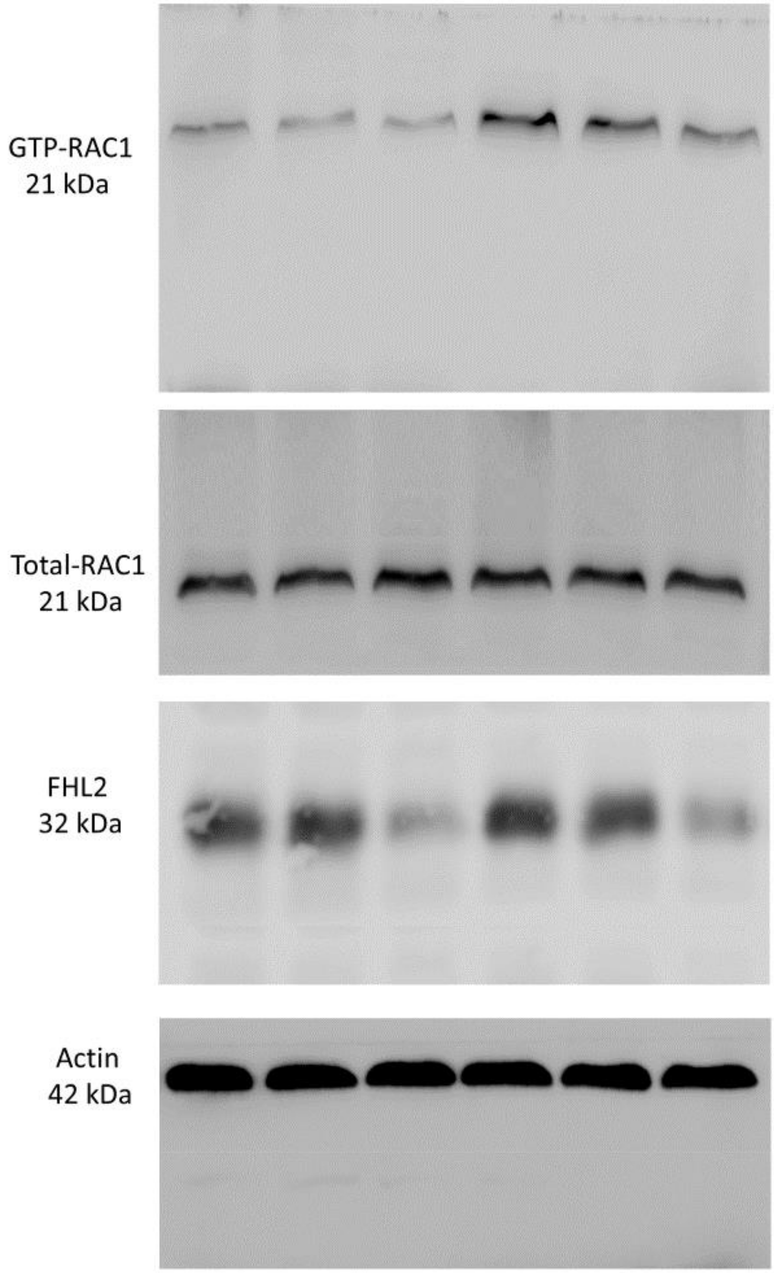

Uncropped western blot gels in figure 5a

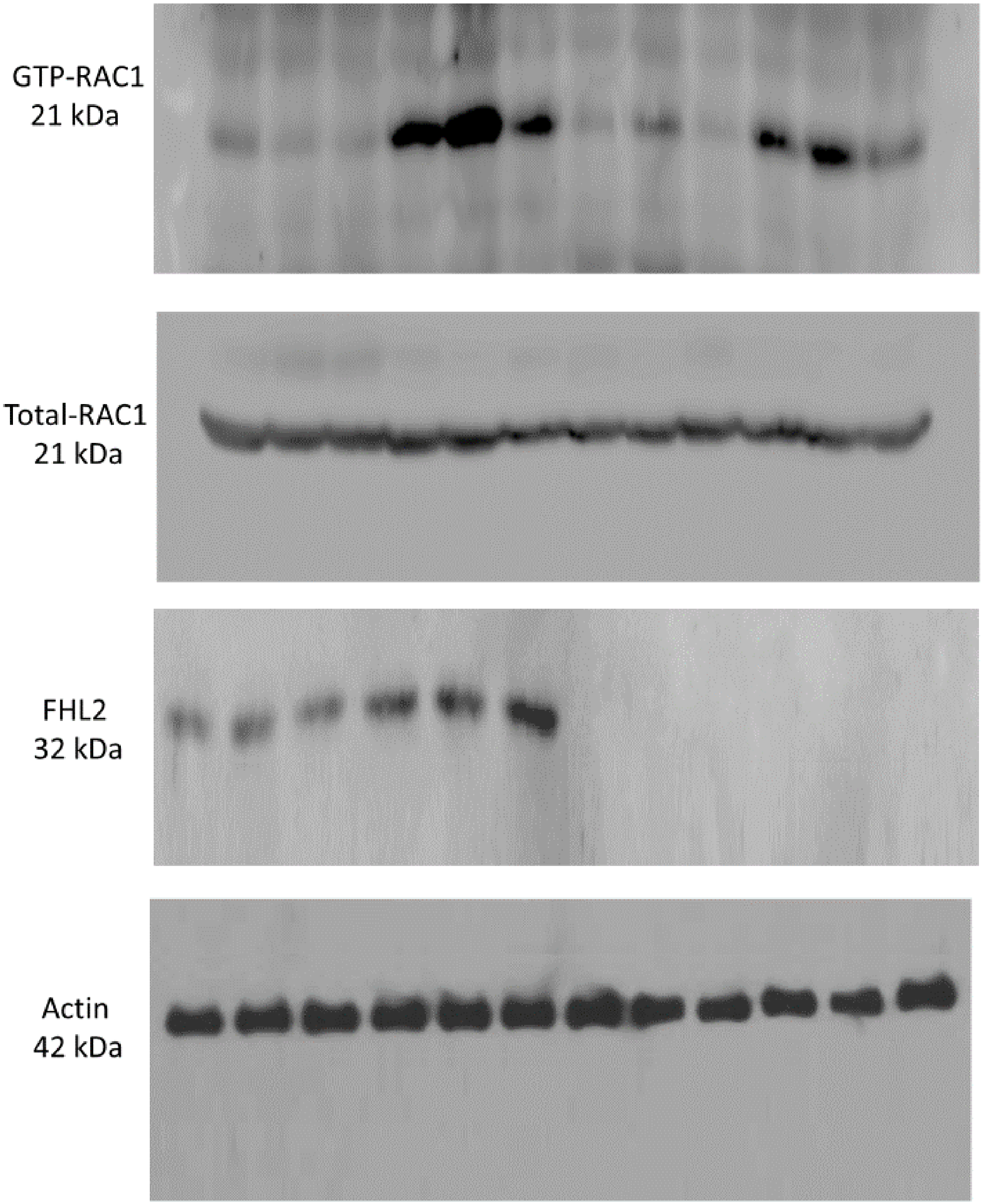

Supplement: Supplementary file 1 — Supplement [file 41598_2019_42328_MOESM1_ESM.pdf]
